# Perinatal Nutritional and Metabolic Pathways: Early Origins of Chronic Lung Diseases

**DOI:** 10.3389/fmed.2021.667315

**Published:** 2021-06-15

**Authors:** Celien Kuiper-Makris, Jaco Selle, Eva Nüsken, Jörg Dötsch, Miguel A. Alejandre Alcazar

**Affiliations:** ^1^Department of Pediatric and Adolescent Medicine, Translational Experimental Pediatrics—Experimental Pulmonology, Faculty of Medicine and University Hospital Cologne, University of Cologne, Cologne, Germany; ^2^Department of Pediatric and Adolescent Medicine, Faculty of Medicine and University Hospital Cologne, University of Cologne, Cologne, Germany; ^3^Center for Molecular Medicine Cologne (CMMC), Faculty of Medicine and University Hospital Cologne, University of Cologne, Cologne, Germany; ^4^Excellence Cluster on Stress Responses in Aging-associated Diseases (CECAD), Faculty of Medicine and University Hospital Cologne, University of Cologne, Cologne, Germany; ^5^Member of the German Centre for Lung Research (DZL), Institute for Lung Health, University of Giessen and Marburg Lung Centre (UGMLC), Gießen, Germany

**Keywords:** lung development and pulmonary diseases, perinatal nutrition, maternal obesity, intrauterine growth restriction, chronic lung disease, bronchopulmonary dysplasia (BPD)

## Abstract

Lung development is not completed at birth, but expands beyond infancy, rendering the lung highly susceptible to injury. Exposure to various influences during a critical window of organ growth can interfere with the finely-tuned process of development and induce pathological processes with aberrant alveolarization and long-term structural and functional sequelae. This concept of developmental origins of chronic disease has been coined as perinatal programming. Some adverse perinatal factors, including prematurity along with respiratory support, are well-recognized to induce bronchopulmonary dysplasia (BPD), a neonatal chronic lung disease that is characterized by arrest of alveolar and microvascular formation as well as lung matrix remodeling. While the pathogenesis of various experimental models focus on oxygen toxicity, mechanical ventilation and inflammation, the role of nutrition before and after birth remain poorly investigated. There is accumulating clinical and experimental evidence that intrauterine growth restriction (IUGR) as a consequence of limited nutritive supply due to placental insufficiency or maternal malnutrition is a major risk factor for BPD and impaired lung function later in life. In contrast, a surplus of nutrition with perinatal maternal obesity, accelerated postnatal weight gain and early childhood obesity is associated with wheezing and adverse clinical course of chronic lung diseases, such as asthma. While the link between perinatal nutrition and lung health has been described, the underlying mechanisms remain poorly understood. There are initial data showing that inflammatory and nutrient sensing processes are involved in programming of alveolarization, pulmonary angiogenesis, and composition of extracellular matrix. Here, we provide a comprehensive overview of the current knowledge regarding the impact of perinatal metabolism and nutrition on the lung and beyond the cardiopulmonary system as well as possible mechanisms determining the individual susceptibility to CLD early in life. We aim to emphasize the importance of unraveling the mechanisms of perinatal metabolic programming to develop novel preventive and therapeutic avenues.

## Introduction

Chronic lung diseases (CLD) such as asthma, chronic obstructive pulmonary disease (COPD) and pulmonary arterial hypertension (PAH) have a major impact on global health, with COPD being the third leading cause of death worldwide (WHO Global Health Estimates, 2020). CLDs do not only have an enormous impact on the patient's quality of life, but also on health care costs (e.g., an average of $4147 per COPD patient per year) (1, 2). While the pathology of adult lung diseases and the influence of environmental factors such as smoking have been extensively studied, the mechanisms determining the individual susceptibility to CLD early in life remain elusive. This review will provide insights in the current knowledge on how perinatal nutritional and metabolic conditions adversely affect lung development and contribute to the origin of CLDs.

Maternal obesity and intrauterine growth restriction (IUGR) represent alterations of the antenatal, perinatal and postnatal nutritional and metabolic status with adverse consequences for the fetus and newborn. (1) First, both maternal obesity and IUGR increase the risk of pregnancy complications and prematurity of the offspring. Epidemiological studies have shown that not only the risk of pregnancy complications for overweight and obese mothers is higher; it is also associated with an early pregnancy loss, congenital malformations, premature birth and stillbirth (3). In addition, the offspring has an increased risk of being either macrosome or IUGR, both introducing their own risk of comorbidity. IUGR is diagnosed in 5–10% of all pregnancies, characterized as a rate of fetal growth less than the growth potential that is appropriate for the gestational age, and well–recognized as an additional risk factor for prematurity (4, 5). (2) Second, fetal and postnatal nutritional supply as well as maternal weight and metabolism can adversely affect the long-term health of the child. This is referred to as *perinatal or metabolic programming* (6, 7). This concept was initially coined by Barker as the *fetal origins* hypothesis, also known as *fetal programming*. Barker et al. proposed that the developing fetus adapts its growth rate and metabolism as a response to variations in the supply of nutrients (and oxygen), which may lead to permanent changes of organs' structure and physiology in the newborn (8). Over the past two decades, the developmental origins of health and disease have gained increasing scientific interest. There has been an enormous effort and an accumulation of studies devoted to elucidating the underlying mechanisms of perinatal (metabolic) programming of diseases as well as its prevention and therapy. (3) Lastly, maternal obesity and IUGR are associated with long-term alterations of lung function and lung structure. For example, clinical reports showed a positive linear trend between birth weight, adjusted for maternal factors, and lung function in adulthood (9). Furthermore, children that were exposed to maternal obesity during pregnancy or gestational diabetes mellitus (GDM) have an increased risk of developing asthma in childhood (10–12). These findings indicate the significant impact of body weight, nutrition, and metabolism during critical phases of pregnancy and the early postnatal period on the lung development and later pulmonary function of a child (13).

**Figure 1:**
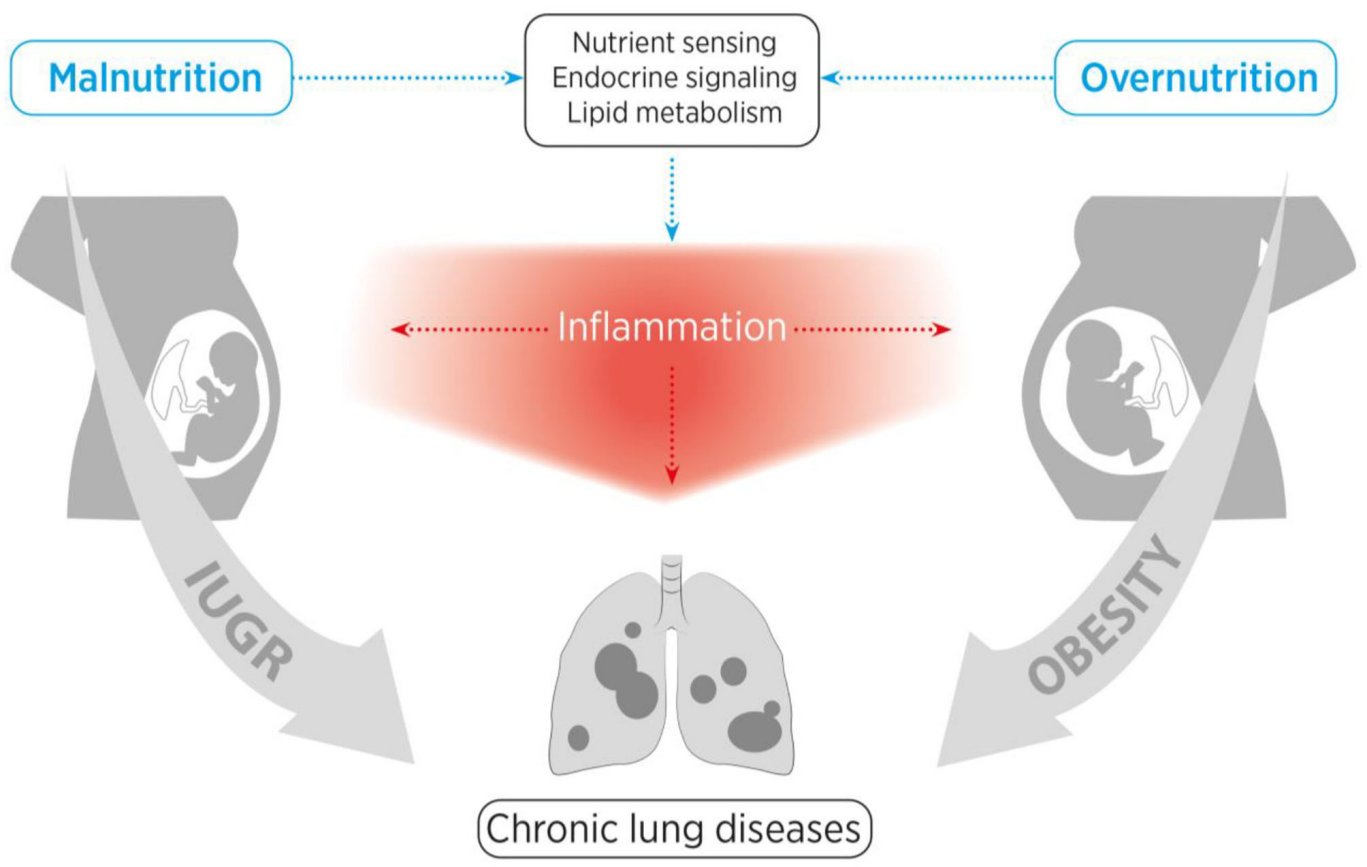
Schematic representation of the structure of this review. We aim to provide a comprehensive overview of the influences of maternal obesity, early childhood obesity and intrauterine growth restriction (IUGR) on nutrient sensing as well as endocrine and inflammatory pathways, and how these adverse perinatal effects contribute to the early origins of chronic lung diseases.

In addition to the adverse nutritive and metabolic influences, the time of exposure is of great importance with regard to the resulting lung pathology. There are different critical windows of lung development with diverse developmental biological processes. The lung develops in five stages, with the last (alveolarization) starting shortly before birth and continuing beyond infancy (14). The window and the nature of exposure to adverse influences render not only the prenatal, but also postnatal lung development highly susceptible to injury and CLDs (15). This basic principle of timing emphasizes the far-reaching complex consequences of antenatal, perinatal and postnatal nutrition. Here, we provide an overview of the impact and mechanisms of nutritive surplus with metabolic disorder (maternal obesity) as well as nutritive deprivation (e.g., IUGR) on the child's lung health (schematic representation in Figure 1).

## The Impact of Perinatal Nutritive Surplus on the Origins of Chronic Lung Disease

Obesity and overweight result from an imbalance of energy consumption and energy intake, causing fat accumulation in adipose tissue (16). The origin of obesity is multifactorial and comprises a complex interaction of genetic and life style factors (17, 18). It is widely accepted that each individual has a certain level of predisposition for obesity due to genetic and epigenetic adaptations along with modifying environmental factors that can in part contribute to familiar obesity (17). Two central endocrine pathways in obesity are those of insulin and leptin. Insulin is a critical regulator of adipocyte biology that promotes the uptake of glucose and fatty acids and stimulates lipogenesis while inhibiting lipolysis (19). In obesity, the glucose transport and adipocyte metabolism are decreased despite high circulating levels of insulin, also known as insulin resistance (20). Leptin is produced by adipose tissue and acts as a regulator of appetite and energy expenditure (21). Obesity is associated with high levels of circulating leptin combined with leptin resistance (22). Leptin and insulin directly interact with each other and in addition, leptin influences insulin sensitivity through the regulation of glucose metabolism (23). Interestingly, targeting the energy balance to favor weight loss might induce compensatory behavioral and metabolic actions that favor the maintenance of bodyweight (24). This is one of the explanations for the further increasing numbers of obesity, despite multiple broad scale attempts on lifestyle and dietary interventions. Instead, obesity has grown into a worldwide pandemic. Surveys conducted by the WHO in 2008 showed that around 1.5 billion adults worldwide suffer from overweight, which corresponds to a body mass index (BMI) of over 25. Of far greater concern are the ~200 million men and 300 million women with a BMI of more than 30, therefore considered to be obese (25). It is alarming that the prevalence of overweight and obesity is not only increasing dramatically among adults, but also children (26).

### Linking Maternal to Childhood Obesity: a Transgenerational Vicious Circle

Early childhood obesity has been proposed as a strong predictor of overweight in early adulthood (27). It has also been reported that maternal obesity and GDM can cause early-onset childhood obesity, which is associated with a higher prevalence of overweight or obesity in adulthood (28). In the USA, 17% of children are already considered obese (26, 29). Studies show that 1 to 2-year-old overweight children are more likely to be obese in their teenage and middle age years, and are prone to develop early-onset metabolic syndrome (30–33). Metabolic syndrome is characterized by obesity, type 2 diabetes, cardiovascular diseases, dyslipidemia and hypertension. As the prevalence of obesity amongst young adults continues to rise, the number of overweight/obese pregnant women is also steadily increasing. Interestingly, there is accumulating evidence of a transgenerational effect of obesity that adversely affects child health throughout life (34, 35). Specifically, maternal BMI shows a significant correlation with high offspring's birth weight and children's overweight (30, 36, 37). Thus, children of overweight mothers are at high risk of developing overweight later in life and tend to suffer from overweight-associated diseases (38). These findings are supported by experimental studies that show higher tendency for obesity and impaired insulin response in offspring of obese dams (39–41).

These transgenerational effects can be attributed, in part, to epigenetic changes in the offspring of obese mothers. The DNA is hypomethylated at the start of embryonic development, therefore, the developing embryo is particularly sensitive to epigenetic changes (e.g., DNA methylation, histone modification and microRNA expression) in response to the intrauterine environment (42). For example, maternal high-fat diet before, during and after pregnancy has been shown to alter miRNA expression and to induce a chronic dysregulation of insulin-like growth factor 2 (IGF-2) signaling in a mouse model (43). Such changes are not only detectable in adult mice (42), but also in human fetal umbilical cord blood (44). In addition, there are multiple reports of modified DNA methylation on sites of importance for metabolic processes after dysglycemia and/or high-fat diet during pregnancy (45–47).

In addition to metabolic consequences, the rising incidence of early childhood obesity is particularly concerning because of the association with respiratory symptoms and diseases in youth. One of the most common respiratory symptoms in childhood is wheezing, with ~30% of all children suffering from it (48). The risk of recurrent wheezing is especially high in children of obese mothers (49, 50). Obese children experience exertional dyspnea and often suffer from obstructive sleep apnea syndrome (OSAS) as well as obesity hypoventilation syndrome (OHS) (51, 52). In case of an emergency, obese children show dyspnea related to sedation and post-operative care (53). Furthermore, persistent asthma is strongly associated with high BMI throughout childhood (54–56), which manifests in a 92% higher prevalence of asthma in adult obese patients (57). In addition, the functional parameters of lung function such as respiratory muscle strength and endurance, airway resistance, lung volume and function or gas exchange are negatively influenced by obesity (58–60). Collectively, these studies indicate an adverse clinical course of respiratory diseases in children of obese mothers and after childhood obesity. In the following, we will discuss possible causes for the association between maternal obesity and child lung health.

### Molecular Insights Into the Mechanical Effects of Obesity on Lung Function

Mechanical and physical influences on the lung play a significant role in the overall health of obese children and adults. Obese persons, including children, experience a lung restrictive syndrome, which goes along with increased overall body volume causing a narrowing of the upper airway and a reduced full inflation due to neck fat and an inadequate thoracic expansion, respectively (61). Accordingly, obese children suffer from obstructive sleep apnea hypoventilation syndrome (OSAHS) which is associated with hypoxemia, hypoventilation, sleep interruptions and chronic fatigue (62). Therefore, OSAHS has a drastic effect on oxygen supply and can induce hypoxia-associated changes in gene expression through the transcription factor hypoxia-inducible factor (HIF) (63). Under normoxia, prolyl hydroxylases (PHDs) hydroxylate proline residues on HIF-α subunits leading to subsequent proteasomal degradation through ubiquitination. In contrast, hypoxia reduces the O_2_-dependent hydroxylation of HIF-α subunits by PHDs, resulting in nuclear HIF-α accumulation (64). In a murine model, the effects of hypoxia-mediated HIF activity have been reported to be involved in the pathogenesis of pulmonary arterial hypertension (PAH), in part by upregulating the vasoconstrictor endothelin-1 (65, 66). Deficiency of HIF-2α, however, partly protected from the increase in endothelin and PAH (65, 66). In addition, stabilization of HIF-α induces alveolar epithelial type 2 cell (ATII) apoptosis and subsequent fibrotic lung diseases (67). In contrast, the use of PHD inhibitors *in vivo* to stabilize HIF-α improved lung growth and function in a model of prematurity (68, 69).

It has been shown that HIF-1α in part sustains the Warburg effect (70, 71). The Warburg effect describes a condition in which the cells obtain their energy mainly through glycolysis with subsequent excretion of lactate. This alternative metabolic state for energy production is used by cancer cells, but also by healthy cells under hypoxia (*anaerobic glycolysis*). As described above, obesity-associated mechanical forces can lead to an activation of HIF, a mediator of hypoxia. HIF can cause a shift toward glycolysis rather than oxidative phosphorylation, in order to meet the energy demands under hypoxic conditions (70). Interestingly, studies suggested a Warburg effect inversion, a condition in which cancer cells exposed to an adiposity environment increase energy production by aerobic respiration as well as gluconeogenesis (72). The authors suggest that the cells do not consume glucose in glycolysis, but produce glucose through gluconeogenesis. Moreover, it has been described that during hypoxia, mitochondria increase the production of reactive oxygen species (ROS) at complex III (73), leading to inhibition of PHD activity and subsequent stabilization of HIF-α (74, 75). The shift toward glycolysis by the Warburg effect and the increased production of ROS, both induced and maintained by hypoxia, resemble mitochondrial dysfunction (76, 77). Increasing evidence points toward a central role for mitochondrial dysfunction in the development of cancer as well as CLDs including asthma, COPD and PAH (71, 76, 77). Furthermore, recent studies have indicated that the hypoxia-induced increase of ROS in acute lung injury contributes to pulmonary fibrosis by triggering an epithelial-mesenchymal transition (EMT) (78, 79) *via* the stabilization of HIF-1α in several cell types, including alveolar epithelial cells (80).

These findings highlight the effect of obesity on oxygen sensing and energy metabolism as well as the subsequent consequences for the development of CLDs. HIF, as a central player in oxygen sensing, might serve as a potential therapeutic approach to target the rising incidences of obesity-related diseases. For example, preclinical data show that blocking HIF with digoxin in a mouse model prevented or slowed down the progression of PAH (81, 82). These promising findings demonstrate that not only preventing obesity itself, but also targeting specific metabolic processes might offer new preventive strategies for CLDs.

### Cell Homeostasis and Inflammatory Response Under Obese Conditions

Obesity represents a state of *low-grade chronic inflammation*. The numbers of inflammatory cells such as CD8^+^, CD4^+^ and CD68^+^ cells are significantly elevated in adipose tissue (83, 84). These immune cells along with adipocytes release a wide range of inflammatory factors including leptin, tumor necrosis factor-alpha (TNF-α) (85), interleukin 6 (IL-6), and IL-8 (86), C-reactive protein, monocyte chemoattractant protein-1 (MCP-1), and Plasminogen activator inhibitor-1 (PAI-1) (87–89). Exposure of the lung to these pro-inflammatory cytokines can occur in three different ways at different time points during lung development: (1) through transplacental transport from the obese mother to the fetus; (2) through breast milk of the obese mother during lactation; and (3) through the child's own adipose tissue as a result of (early) postnatal obesity. For example, maternal high-fat diet in a murine model during lactation [postnatal day 1 (P1) to P21] induced an early-onset obesity in the offspring with elevated inflammatory cytokines, such as IL-4, IL-6, IL-13, IL-17A, and TNF-α. The early inflammatory response was related to increased airway hyperreactivity, similar to asthma (90). The adverse effect of IL-6 on the lung was further supported by a study that showed that elevated IL-6 could in part account for the development of emphysema through IL-6 trans-signaling-mediated apoptosis of ATII. Blocking IL-6/gp130 signaling, however, prevented features of lung emphysema (91, 92). Furthermore, elevated IL-6 levels contribute to PAH (93). For example, IL-6 induces a downstream activation of Stat3, which in turn causes a phosphorylation of the transcription factor forkhead box O (FoxO) 1. Phosphorylation of FoxO1 leads to its cytoplasmatic sequestration, subsequent inactivation and ultimately to a hyperproliferation of bronchial smooth muscle cells (SMC) (93, 94). In addition to IL-6, TNF-α is also a notable adipocytokine that is elevated under obese conditions (95). TNF-α modulates the effects of G-protein coupled receptor (GPCR)-induced hyperreactivity in cultured murine airway SMCs and increases contractility (96). By this mechanism, TNF-α may be contributing to SMC responsiveness and the development of asthma. Consequently, the anti-inflammatory adiponectin reduces TNF-α-induced nuclear factor κ B (NFκB) signaling. Thus, the obesity-related decrease in adiponectin further contributes to a dysregulated TNF-α signaling (97). Due to its potential impact on the development of asthma, TNF-α is under intense investigation as a therapeutic target (98–100). Another important functional aspect of TNF-α is the ability to contribute to insulin resistance by inhibiting tyrosine phosphorylation of insulin receptor substrate-1 (IRS-1) (95). Similarly, PAI-1 is produced and secreted by adipocytes and elevated in obesity serum levels (89). In a mouse model of airway hyperresponsiveness, PAI-1 was involved in airway remodeling after LPS-induced lung injury (101). Chronically elevated levels of PAI-1 affect the extracellular matrix turnover and contribute to collagen deposition in the airways (102). Moreover, dysfunction of the adipose tissue after perinatal obesity can further contribute to the maintenance of *low-grade chronic inflammation*. For example, a recent study indicated that maternal obesity induces metabolic programming of adipocytes in the offspring with lifelong dysfunctional adipose tissue and obesity (103). Collectively, obesity represents a state of *low-grade chronic inflammation* exposing the developing lung to pro-inflammatory cytokines which could adversely affect lung growth as a first “hit” and increase susceptibility for CLDs in later life.

### Nutrient Sensing and Leptin Signaling as a Mechanism of Perinatal Obesity

Under physiological conditions, leptin is integrated in the complex mechanisms of airway and bronchial maturation. A recent study highlighted the importance of physiological non-obese levels of leptin in lung maturation through the upregulation of the expression and the secretion of surfactant protein A (*Sftpa*) in ATII (104, 105). Similarly, leptin promoted maturation of lung structure and contributed to postnatal lung remodeling and enlargement of the alveolar surface area *via* the induction of the genes *Col1a1, Col3a1, Col6a3, Mmp2, Tieg1*, and *Stat1* (106). A lack of leptin signaling in *ob/ob* mice (induced by leptin deficiency) resulted in a significant reduction of alveolar surface, indicating a critical role of leptin in postnatal lung growth (106). These contradictory observations may be due to effects of high circulating concentrations of glucose and insulin during pregnancy in obese mothers, which might potentially overrule the beneficial effect of leptin on lung development (107). In addition, long-term exposure to leptin before birth could affect the expression of pulmonary leptin receptors, disturbing leptin-signaling, leading to defective lung maturation and respiratory function at birth (108, 109).

Leptin has a central role in the immune response as well. Leptin was linked to asthma in adults as well as in children; the severity of asthma was correlated to serum leptin levels in a meta-analysis of 13 studies (110–113). High leptin levels increased the T-helper cell type 2 (Th2)-type immune response in airways *via* a leptin-mediated and XBP1 (X-box binding protein 1) s-dependent activation of mTOR (mechanistic target of rapamycin) as well as MAPK (mitogen-activated protein kinase) signaling (114). A shift toward the Th2-type immune response in airways is characteristic for the pathogenesis of asthma, thus providing a relevant link between obesity-induced high circulating leptin levels and the development of asthma (114, 115). Adiponectin acts as an anti-inflammatory agent, counteracting leptin (88, 89). Circulating adiponectin levels are known to be reduced in obesity, possibly further contributing to the pathogenesis of obesity-associated asthma (116).

There have been several attempts to alter the high leptin and low adiponectin levels in order to restore the metabolic balance. For example, pharmacological elevation of adiponectin levels in obese mice protected from hyperglycemia, glucose intolerance, and insulin resistance (117) as well as increasing insulin sensitivity (118). However, to date, the effect of adiponectin supplementation on pulmonary development and function remains elusive. In diabetes, thiazolidinedione (TZD) is possibly the most extensively characterized regulator of adiponectin expression. TZDs, such as pioglitazone and rosiglitazone increase adiponectin expression through the activation of peroxisome proliferator-activated receptor gamma (PPARγ) (119, 120). Since it is already a well-established therapeutic intervention for diabetes, targeting adiponectin might be a new promising therapeutic approach for the prevention of long-term consequences of obesity such as pulmonary remodeling and reduced lung function.

### Lipid Metabolism and Perinatal Obesity

Obesity is characterized by a dysregulation of the energy and lipid metabolism. Lipoproteins are responsible for the transport of fatty acids, cholesterol and phospholipids. Therefore, the lipoproteins in obese patients show a change in circulating protein levels (121). For example, apolipoprotein E (ApoE), which is part of the low-density lipoprotein (LDL), is elevated in the obese and contributes to fat mass accumulation (122). LDL/ApoE is internalized into cells by its receptor, the low-density lipoprotein receptors (LDLRs), and is the main source of cholesterol and phospholipids efflux out of cells. In the lung, ApoE is produced by lung macrophages and acts on ciliated airway epithelial cells, where it can modulate airway hyperreactivity, mucin gene expression, and goblet cell hyperplasia (121). Thereby, it is involved in reducing the susceptibility to airway hyperresponsiveness (121, 123). In line with this, genetic modified mice with an ApoE deletion show reduced alveologenesis and abnormal pulmonary function with increased airway resistance as well as high dynamic and static compliance (124). PPARγ is a nuclear receptor and considered one of the master regulators of adipogenesis, showing a high expression pattern in adipose tissue and in the lung (125–127). PPARγ is essential for normal lung development *via* the induction of alveolar epithelial-mesenchymal paracrine signaling (128, 129). Murine studies with genetically deactivated PPARγ demonstrated a spontaneous development of PAH. Here, PPARγ has an anti-proliferative effect on smooth muscle cell proliferation, which might give the opportunity to use PPARγ agonists in treating PAH (130, 131). Moreover, unsaturated fatty acids and several eicosanoids are regulators of PPARγ and induce expression of genes encoding lipoprotein lipase, CD36, phosphoenolpyruvate carboxykinase, aquaporin 7 and adiponectin (132). This is of particular interest since the western style diet has high concentrations of poly-unsaturated fatty acids (133). In this context of western style diet and a higher rate of obese individuals in industrial western countries, elevated fatty acid levels in obesity may be important regulators and modulators of normal and aberrant lung development.

### Glucose Metabolism and Hyperinsulemia

Obesity is intimately linked to insulin resistance, accompanied by elevated circulating insulin concentrations. The transduction of insulin signaling is in part mediated through the downstream phosphatidylinositol 3-kinase (PI3K)/protein kinase B (AKT) and mTOR pathways (134–137). The mTOR cascade is integral in orchestrating the complex mechanism of lung development, balancing nutrient and energy supply in the early stages of embryogenesis and fine-tuning tissue growth during organogenesis. An elaborated and comprehensive article by Land et al. provides a broad overview of the role of mTOR in lung development (138). High levels of insulin from diabetic mothers have the potential to inhibit the *Sftpa* gene expression in lung epithelial cells and thereby delay the fetuses' lung development. This insulin-induced inhibition acts *via* the rapamycin-sensitive PI3K signaling pathway and not *via* mitogen-activated protein kinase (MAPK) (139). This notion is further supported by the fact that inhibition of PI3K can contribute to insulin resistance and diabetes (140). Moreover, Ikeda and colleagues demonstrated that insulin reduces vascular endothelial growth factor (VEGF) expression and the transcriptional activity of HIF-2 on the VEGF promoter in an AKT-mTOR-dependent manner in cultured lung epithelial cells. They further demonstrated that activation of the AKT-mTOR pathway in mice reduced alveolar capillarization, stressing the importance of this pathway in lung epithelium and in the development of infant respiratory distress syndrome (RDS) (141). Interestingly, moderate physical activity of obese mothers can rescue maternal and the offsprings' insulin sensitivity, overall improving the metabolic, as well as potential pulmonary outcome in the obese mother as well as her offspring (142).

Insulin does not only affect the alveolar epithelial cells, but also increases the expression of genes related to the contractile phenotype of airway SMC through a Rho kinase- and PI3K-dependent mechanism (143). Apart from these direct effects on pulmonary cells, insulin is involved in the modulation of the immune response and thereby in the pathogenesis of asthma. For example, in mast cells, insulin induces PI3K-dependent signaling, which could contribute to allergic bronchoconstriction (144). On the other hand, Viardot and colleagues demonstrated that insulin influences T cell differentiation promoting a shift toward a Th2-type response. They state, that this effect may contribute to insulin's anti-inflammatory role in chronic inflammation associated with obesity and type 2 diabetes (145). Insulin further exhibits anti-inflammatory properties in acute Th1-type inflammation, where insulin diminishes acute lung injury and reduces levels of inflammatory cytokines (146). Taken together, insulin plays an important role in physiological lung development, supporting alveolarization. In obese patients, however, elevated insulin levels interfere with lung development and maturation, while facilitating a pro-asthmatic immune environment, which could affect the outcome of CLDs in later life.

Collectively, these studies show that perinatal obesity resulting from maternal and early childhood obesity may determine individual susceptibility for CLDs later in life. In addition to mechanical factors due to increased body mass, adipose tissue dysfunction and its consequences play a particularly important role. *Low-grade chronic inflammation* with increased levels of adipocytokines, impaired insulin signaling, and altered lipid metabolism can be important in metabolic programming of CLDs. In the future, further elucidation of the fat-lung axis is imperative for a better understanding of metabolic mechanisms in the development of CLDs and to develop new preventive and therapeutic approaches.

## The Impact of Perinatal Nutritive Deficiency on the Origins of Chronic Lung Disease

### Nutrient Deprivation and Lung Development: the Role of Intrauterine Growth Restriction

Intrauterine growth restriction (IUGR) was first described as “dysmaturity” and indicates an abnormally low birth weight for the gestational age. Classically, IUGR was defined as a birthweight below 2,500 g (147). More recently, it has been characterized as “not reaching the biologically based potential,” often due to reduced perfusion or malnutrition *in utero* (148–150). IUGR and “small for gestational age” (SGA, birthweight of-2 SD/mean) are often used interchangeably; however, SGA neither excludes nor proves IUGR but serves as an easily quantifiable proxy for IUGR. Pathological intrauterine circumstances induce IUGR, resulting in an infant with low birth weight, often followed by a period of rapid postnatal weight gain, also called “catch-up growth.” Catch-up growth is associated with altered nutrient supply, and overlaps with the final stages of pulmonary alveolarization and vascular maturation (151, 152). Moreover, infants with catch-up growth after IUGR have a higher risk to become overweight or obese and to develop metabolic disorders later in life (4, 153). These clinical findings have been supported by experimental models of IUGR (154–157).

The etiology of IUGR can be divided in (1) *fetal origins*, such as genetic abnormalities (e.g., chromosomal abnormalities), (2) *maternal factors* (e.g., vascular diseases, persistent hypoxia or undernutrition, and toxins), and (3) *placental etiologies* (e.g., placental insufficiency, inflammation) (158). It is thought that 40% of birth weight is ascribable to genetic factors and that the remaining 60% is due to fetal environmental exposures (159). Several historical events have caused a surge of IUGR cases in a defined birth cohort, which has provided deeper insight into the clinical sequelae of IUGR. The latest temporary surge of IUGR caused by maternal malnutrition in Europe was caused by the Second World War. Investigations of the Dutch Famine Birth Cohort (Amsterdam, 1944-1946) have shown that low birth weight infants often have a lower FEV1 and FVC, but not FEV1/FVC ratios, indicative of restrictive lung alterations (160, 161). Other cohorts, however, including an Indian study demonstrate an association of small head circumference (indicative of early gestational growth restriction) with reduced FEV1/FVC ratios (162, 163). These data show the diverse impact of intrauterine nutrient deprivation on lung health that could be in part accounted to the window of injury or the type of nutrient restriction (e.g., protein, vitamins). Overall, these observational and experimental studies highlight that being born IUGR represents a pathologic condition with far-reaching consequences for the child's health and disease, especially regarding metabolism and the lung.

### The Interplay Between IUGR and Obesity

Maternal obesity and GDM are often associated with macrosomic offspring (164). However, in uncontrolled or badly controlled GDM, diabetic vasculopathy and nephropathy may lead to placental insufficiency-induced IUGR (165, 166). In addition, experimental data have shown that overnutrition of pregnant sheep causes IUGR in the fetus, likely due to major restriction in placental growth and relative hypoglycemia and fetal hypoinsulinemia during late pregnancy (167). This might be partly related to fetal hypoxia, in turn inducing fetal catecholamine expression and reducing circulating insulin concentrations (168). On the contrary, IUGR induces metabolic changes to the growing fetus that cause a risk for developing obesity, diabetes and metabolic syndrome later in life (4, 153). These changes are passed onto the next generation; female IUGR rat offspring exhibit symptoms of gestational diabetes, and their offspring has increased fasting glucose and insulin levels despite having a normal birth weight when compared to controls (169). These transgenerational changes might be attributed to epigenetic changes, not only affecting the IUGR offspring, but also the second generation by direct exposition of the offspring germ-line to the IUGR environment (170, 171). More specifically, the increased risk for childhood and adult obesity in IUGR offspring could be in part due to *programming* of the adipocytes toward lipogenesis and proliferation (172, 173). Moreover, the combination of IUGR (induced by surgical bilateral artery ligation) with maternal obesity increased hepatic cholesterol accumulation and LDLR expression when compared to non-IUGR controls (156). These data further support the notion that maternal obesity along with IUGR provides an additional risk for metabolic complications.

### The Adverse Effects of IUGR on Pulmonary Structure and Function

IUGR causes structural changes to the lung. Multiple animal studies have shown that IUGR impairs alveolar formation and lung growth, leading to reduced lung function (155, 174–179). In addition, a recent study from our group has demonstrated that IUGR also negatively influences angiogenesis and extracellular matrix formation (157). The intimate link between angiogenesis and alveologenesis has been shown in various animal studies, where alveolar formation was reduced after blocking angiogenesis (180–182). Conversely, the positive influence of angiogenesis on alveolar growth and regeneration is of great therapeutic importance (181, 183). Structural alveolar and vascular changes during lung development could account for the functional alterations that were reported after IUGR in epidemiological studies: several cohort studies have shown that school-children born IUGR have a significantly lower FEV1 and airway resistance as well as a higher susceptibility to airway infections, independent of catch-up growth (184–189). Moreover, in long-term follow-up studies it was shown that a low birthweight decreases lung function in adulthood, with a reduction of lung capacity and elasticity, resembling a COPD phenotype (9, 190). In summary, there is compelling epidemiological and experimental evidence that IUGR determines lung structure and function and could thereby predispose for CLDs.

### Endocrine Effects of IUGR and Catch-Up Growth Resemble those of Obesity

Children born SGA have an increased risk of reduced embryonic β-cell growth, glucose intolerance, insulin resistance, type II diabetes and obesity in childhood as well as later in life (191–197). The effect of IUGR on the regulation of insulin levels has been extensively studied, as insulin is not only important for euglycemia in the fetus but also serves as a major fetal (pulmonary-) growth factor (159). The stable glucose flow over the placenta during healthy pregnancy causes fetal insulin secretion that regulates normal adipose tissue development and deposition (198). As stated before, IUGR fetus can exhibit hypoglycemia and hypoinsulinemia due to inhibition of endocrine signaling by catecholamines (168). In addition, the pancreatic function can be decreased after IUGR, resulting in lower levels of intrauterine insulin secretion as well (199). In contrast, reports on postnatal insulin levels in IUGR newborns are contradictive, they might be slightly lower or equal to healthy controls (200, 201). Thus, IUGR causes a deregulation of intrauterine insulin levels, an important mediator in adipose tissue development and fat deposition.

The phase of catch-up growth after IUGR appears to be a strong determinant of future (lung) health. A key fetal adaptation to nutrient deprivation is the intrauterine upregulation of the insulin receptor under hypoinsulinemic circumstances in fetal skeletal muscle (202). After birth and under nutrient surplus, this upregulated receptor is activated by an abundance of glucose and insulin, inducing accelerated body growth (202, 203). The closely related insulin-like growth factor 1 (IGF-1) is induced by growth hormone (GH)/somatropin and is an essential regulator of body growth. The inhibition of the GH/IGF-1 axis has been shown to dysregulate alveologenesis, mainly through disruption of the physiological deposition of the extracellular matrix (204). Work by our group has shown that inhibition of the GH/IGF-1 axis by IUGR was associated with an arrest of lung development; in contrast, catch-up growth caused a significant increase of GH/IGF-1 expression (174). Interestingly, recent work demonstrated that postnatal treatment with recombinant human IGF-1 improves lung growth and structure in a model for bronchopulmonary dysplasia (BPD) (205). In conclusion, there is a postnatal reactive upregulation of both the insulin receptor and the insulin-signaling (including IGF-1) pathway after IUGR, resulting in an initially increased insulin sensitivity during postnatal catch-up growth (4). However, school-aged and adolescent children with accelerated weight gain and catch-up growth after IUGR show increased levels of insulin and reduced insulin sensitivity, indicating the long-lasting effects of prenatal metabolic programming (206, 207).

In addition to the dysregulation of prenatal and postnatal insulin signaling, leptin has been identified to be dysregulated after IUGR as well. Animal studies have shown that IUGR rat pups rapidly develop leptin resistance during their catch-up growth, thereby stimulating weight gain through hyperphagia (208–210). An important molecular link between nutrient status, insulin/leptin signaling and metabolic outcome is the mTOR pathway, controlling cell growth in response to its environment (e.g., stress, oxygen, nutrient status) through protein synthesis as well as lipid, nucleotide, and glucose metabolism (211, 212). A study from our group has shown that nutrient sensing *via* the mTOR signaling pathway is dysregulated in lungs from a rat model of nutrient deprivation-induced IUGR (157). Recent reports demonstrated that the mTOR signaling pathway is also altered in the placenta of humans and in experimental IUGR studies, enforcing adaptive mechanisms from both the maternal nutrient supply and the fetus's energy demands (213, 214). These studies suggest that both the placenta and the fetus react to nutrient availability by regulating this key nutrient sensor. The mTOR pathway is postnatally essential for pancreatic β-cell and islet maturation (215). Furthermore, mTOR is a potent mediator of endocrine responses, translating signals from leptin and insulin to a negative feedback for insulin (216). Interestingly, studies demonstrate that mTOR is involved in lung development as well, by regulating cell growth for proper organ development (211, 212) and by interfering with essential developmental signaling pathways, such as pulmonary angiogenesis (VEGF) and extracellular matrix deposition (bone morphogenetic protein, BMP) (217, 218). Collectively, these data highlight the eminent impact of intrauterine nutrient deprivation on endocrine function. Of note are the converging similarities between IUGR and obesity with regard to the endocrine system and the long-term metabolic and pulmonary sequelae.

### IUGR Causes Transgenerational Metabolic Programing

Epidemiological studies as well as animal studies have shown transgenerational effects of IUGR on metabolic function (219–221). In part, these effects can be attributed to epigenetic programming (222, 223). For example, Fu et al. as well as Tosh et al. described histone modification along the IGF-1 gene and subsequently altered mRNA expression of IGF-1 in a rat model for IUGR induced by placental insufficiency or maternal malnutrition, respectively (224, 225). In addition, Tosh et al. showed that the restriction of early postnatal nutrient intake partly prevents these epigenetic changes (224). Park et al. observed consistent epigenetic adaptations related to differential binding of dinucleotide methyl transferase 1 and 3a together with changes in histone acetylation and methylation in the promoter region of the *Pdx1* homeobox gene in a rat model of IUGR (226). Recent studies by Gonzalez-Rodriguez et al., demonstrated the genetic imprinting of H19/IGF2 in second-generation IUGR offspring. This genetic imprinting was associated with altered H19 and IGF2 expression, which is in turn related to an increased risk for obesity and associated metabolic diseases (220, 227). Interestingly, this effect is reversible with postnatal essential nutrient supplementation (220, 228). These studies highlight the influence of perinatal nutrition in the development but also the primary prevention of metabolic diseases, including their secondary pulmonary complications as described in the previous chapter. In summary, these data indicate the great potential of perinatal nutrition and metabolism as a preventive and therapeutic target for metabolic health and CLDs.

### Chronic Inflammation in IUGR-Associated CLD

One of the vital connections between the metabolic consequences of intrauterine nutrient deprivation and altered lung development is chronic inflammation. Chronic inflammation has been associated with (1) IUGR (229–232), (2) obesity, type 2 diabetes and metabolic syndrome (233–235) as well as (3) CLDs (236–239). IUGR, followed by catch-up growth, shows similar endocrine dysregulation and activation of inflammatory mechanisms as obesity. For example, both obesity and IUGR exhibit similar levels of leptin and insulin resistance in response to their prenatal nutritional status and postnatal accelerated weight gain (191–197). A possible shift of the Th2 immune response might be another link between CLDs and metabolic changes, e.g., elevated leptin (240) and insulin (206, 207) levels after IUGR. Interestingly, a study in IUGR mice has shown that the Th2 shift and consequent recruitment of macrophages cause inflammation in the pancreatic β-cell islets, causing type 2 diabetes (230). To date, there is no conclusive evidence whether IUGR-associated chronic inflammation is causative for or a consequence of metabolic distress, but the reports support an intimate link between both conditions.

A clinical study on the cord blood of 20 SGA neonates showed that IUGR causes a low-grade inflammatory response: infants born IUGR had significantly increased levels of inflammatory markers IL-6, TNF-α, CRP and thrombopoetin (232). Moreover, animal studies have demonstrated IUGR-associated systemic inflammation in various organs: adult (uteroplacental) IUGR rats exhibited increased pancreatic β-cell inflammation, increasing the risk of diabetes (230, 241); a sheep model for hypothermia-induced IUGR showed a decrease of NF-κB as key regulator of immune-responses (231); and finally, a recent study in IUGR lambs demonstrated increased inflammatory markers and expression of inflammatory as well as pro-apoptotic genes in liver tissue (229). Along with these reports, prior work from our group has shown that IUGR causes dysregulation of key developmental signaling pathways such as NPY(neuropeptide Y)/PKC(protein kinase C), IL-6/AMPKα and TGFβ (transforming growth factor β) signaling as well as the associated inflammatory response (178, 179, 242).

A highly relevant comorbidity for IUGR infants is prematurity. About 30–50% of all extremely premature infants display symptoms of IUGR (243, 244). The causes of prematurity are multifactorial, but there is a strong correlation with maternal obesity. A meta-analysis of 84 clinical studies has shown a significantly increased risk of (induced) preterm labor in overweight and obese pregnancies (245). In addition, the risk of neonatal respiratory complications after premature birth is higher in obese *vs*. non-obese pregnancies (246, 247).

Premature birth and perinatal inflammatory responses have been intimately linked to pathological processes (248). The lungs of preterm infants are often in the late-saccular to early-alveolar phase at birth and require respiratory support (249). Mechanical ventilation, continuous positive airway pressure (CPAP) or oxygen supplementation are necessary treatments, but cause inflammation, acute lung injury and lead to a neonatal CLD, also known as BPD (250–254). Lungs of infants with BPD are characterized by vascular and alveolar hypoplasia (255). As stated previously, IUGR alone adversely affects lung microvascular and alveolar formation. Interestingly, the combination of IUGR with the immature lung in premature infants increases the risk for the clinical manifestation of BPD (i.e., prolonged need of oxygen supplementation >36 weeks of gestation) (251, 256). These reports indicate that IUGR might be an initial “hit” to the organism, raising susceptibility to CLDs such as BPD.

**Figure 2:**
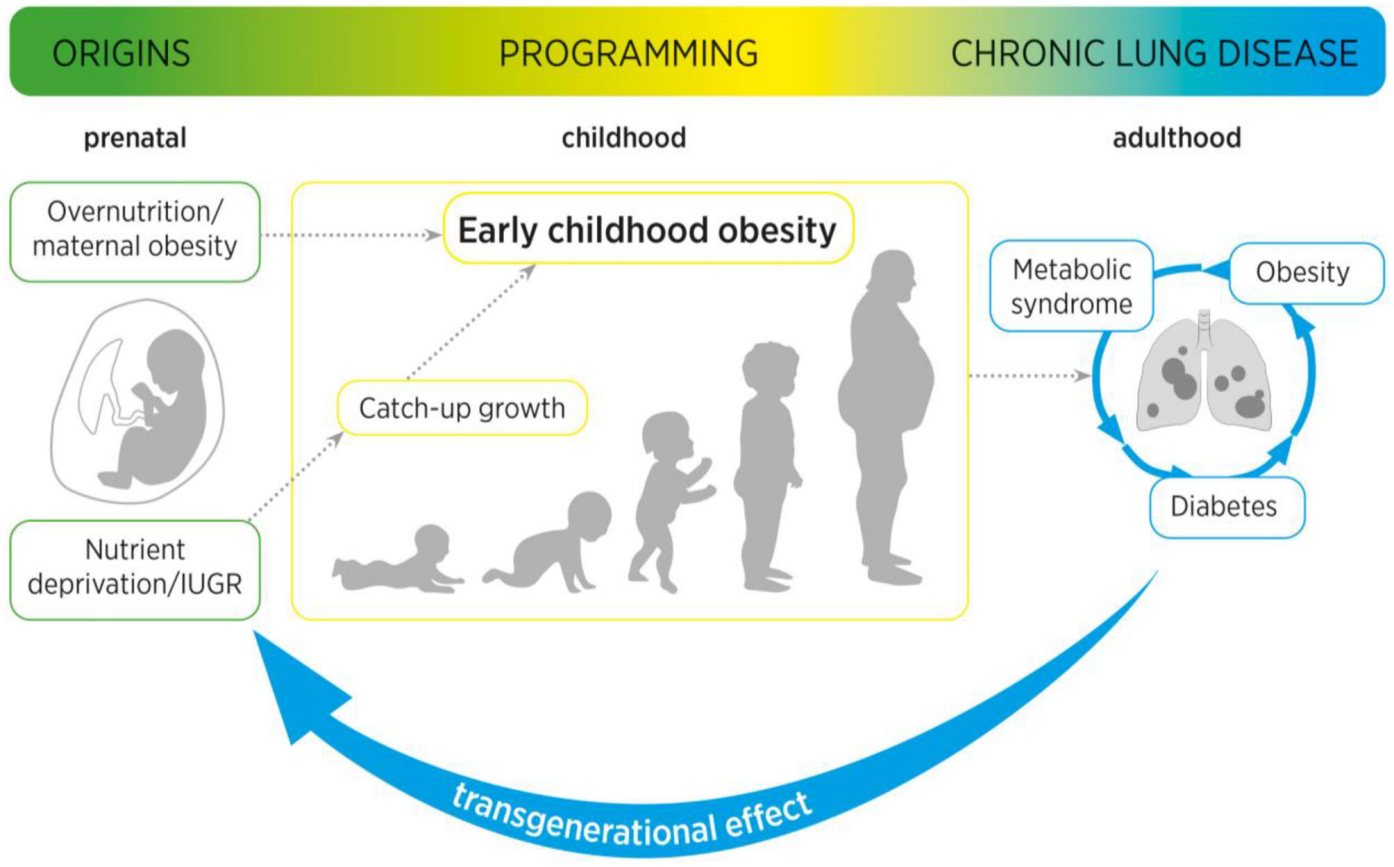
Maternal obesity as well as intrauterine growth restriction (IUGR) increase the risk of (catch-up growth mediated) early childhood obesity. Maternal and early childhood obesity are associated with long-term adverse metabolic effects, including type 2 diabetes mellitus and metabolic syndrome. These pathological metabolic processes are not only intimately linked to an increased risk for pulmonary diseases, but can cause a transgenerational effect from mother to child, to second-generation offspring.

In conclusion, similar to perinatal obesity and GDM, IUGR leads to acute as well as long-term functional and structural changes in the lung. A distinction must be made between an intrauterine and a postnatal phase in the process of perinatal programming caused by IUGR. While the intrauterine phase is characterized by nutritional deprivation, the postnatal phase is usually characterized by a catch-up growth. With regard to the pathomechanisms, metabolic signaling pathways, inflammation, and nutrient-sensing processes play an essential role, ultimately controlling alveolar and vascular formation and lung growth. However, the different phases of injury in IUGR also provide windows of opportunity for preventive strategies, therapeutic interventions and reprogramming in the future (Figure 2).

## The Microbiome as a Link Between Nutrition and Lung Health: Opportunity for Intervention

As stated before, obesity represents a state of *low-grade chronic systemic inflammation*, as illustrated by the increased amount of circulating inflammatory cells (83, 84) as well as elevated expression levels of inflammatory factors (85–88). External influences on the lung health such as airway pollution and cigarette smoking have been extensively studied in the last decade. Recently however, another field of interest has gained momentum: the gut-lung microbiome. This represents an extremely important link between nutrition, chronic inflammation and pulmonary health. In the following, we will detail the role of the microbiome in the early origins of CLDs.

The infant's microbiome is predominantly established during and shortly after birth, where it is exposed to the maternal and environmental microbiome (257). The introduction of solid foods into the children's diet is the next essential step in the microbiome development. Western diet (rich in meat and fat) has been linked to decreased bacterial gut richness, whereas a diet-based on fruits and vegetables is associated with increased bacterial richness (258, 259). The human microbiome is closely related to the nutritional status and chronic inflammatory processes of the individual (260, 261). Studies have shown that it is possible to predict if an individual is lean or obese based on a classification of the gut microbiome with an accuracy of over 90% (262). In humans for example, the abundance of bacteria of the taxa *Christensenella* is negatively correlated with BMI; in contrast, in *in vivo* experiments feeding mice *Christensenella* bacteria induces weight loss (263). Another human study revealed that the gut microbiome can influence leptin concentrations, indicating that the microbiome might regulate appetite (264). Interestingly, it has been shown that the same dietary ingredients have different effects on the blood glucose levels in humans, which is thought to be mediated by the microbiome as well (265). In addition, new studies have shown that the fecal transplantation of lean to obese patients improves insulin sensitivity (266).

The microbiome alters the immune system and future immune response. For example, it has been shown that the yeast *Candida Albicans* in particular has a prominent effect on the TNF-α response of the host; and the palmitoleic acid metabolism of bacteria has been associated with lower systemic responses (267). An overall decrease of bacterial richness is linked to a variety of diseases including obesity, coronary vascular disease, metabolic syndrome insulin resistance, dyslipidemia, and inflammatory disorders (268, 269). When the development of the infants' microbiome is perturbed by the use of antibiotics it can lead to the development of obesity or asthma in later life (270). These examples highlight the mutual relationship between the immune system and the microbiome, creating a finely tuned balance (271). As a result, an imbalance between both creates a lifelong signature of the infants' microbiome (272).

The gut microbiome has been extensively studied, but the lung microbiome has only recently gained interest with the first reports of altered microbiome in asthma (273). The lung microbiome has a strong influence on the susceptibility to a wide array of chronic lung diseases, including COPD, asthma, Idiopathic Pulmonary Fibrosis (IPF) as well as altering the prognosis of cystic fibrosis (CF) (274). In healthy individuals, the lung microbiome is well-regulated by the environment (high clearance, low immigration and low nutrient availability). However, processes that favor alterations of the microbiome and inflammation include the increased production of mucus, creating a moist and warm bacterial niche, increased vascular permeability which increases the nutrient availability and selective growth promotion as well as selective clearance due to the altered immune response to airway colonization (275, 276). These factors promote the bacterial colonization of the airways as well as the selective overgrowth of certain well-adapted species, thereby creating a shift of the microbiome from healthy to diseased and inducing the “dysbiosis-inflammation cycle” as introduced by Dickson et al. (275–278). Thus, a perpetual cycle of microbial changes, possibly due to initial nutritional changes before and early after birth, along with inflammation has a significant impact on the development and prognosis of CLDs.

To date, the gut-lung axis remains elusive, especially with regard to clinical interventions. Nonetheless, several initial successes have been reported in the recent years. For example, stimulation of the gut microbiome with a high-fiber diet in COPD patients has been shown to increase the production of anti-inflammatory short chain fatty acids (SCFAs). These anti-inflammatory factors might reduce chronic inflammation of the lungs, prevent or decrease lung remodeling and therefore improve the lung health of COPD patients (279). Meanwhile, the Canadian Healthy Infant Longitudinal Development (CHILD) Study revealed that bacterial genera *Lachnospira, Veillonella, Faecalibacterium*, and *Rothia* are significantly reduced in infants at risk for asthma. Inoculation with these four bacteria reduced airway inflammation in a mouse model, possibly lowering the risk for asthma (280). These studies highlight the promising benefits of dietary changes or adjustment of the gut microbiome for the improvement of lung health.

The virome, including the genes of pathogenic viruses, resident viruses and bacteriophages, is of interest for CLDs as well (281, 282). Viral infection is the predominant reason for acute respiratory infections and the exacerbation of CLDs such as asthma, COPD and CF (283, 284). Next generation sequencing has made it possible to assess the viral DNA or RNA load in respiratory samples (285, 286). Obesity influences the virome of the host; it has been reported that increased viral RNA abundance is closely correlated to an increase of fat mass and hyperglycemia in mice (287). In line with this finding, obese patients show a higher susceptibility to dengue fever (288). Moreover, the adenovirus Ad-36 interferes with adipocyte differentiation, leptin production and glucose metabolism (289). Special interest is drawn to the fact that viral presence in the gut influences the host's immune response (290) by interfering with the host's microbiome and immune-modulatory actions (e.g., through the TNF-α pathway) (267, 291). This crosstalk between the bacterial microbiome and virome and their immune-modulating properties have also been reported in the lung (277, 292).

In conclusion, nutritional changes can directly and indirectly influence the intestinal and pulmonary microbiome (including the virome), modulating the immune response and increasing inflammation, and ultimately the risk of severe CLD. Targeting the microbiome might offer new preventive and therapeutic avenues for CLDs early in life.

## Systemic Consequences of Fetal Programming

Maternal obesity, maternal malnutrition or fetal nutrient deficiency through placental insufficiency naturally not only affect the lung, but all other organs as well. The organ-specific susceptibility to metabolic influences varies, and the “window of exposure” plays a crucial role. In other words, the timeframe during which organ development and/or -function is especially vulnerable differs from organ to organ. While a lot of research has been performed with traditional technologies, the ever-growing possibilities of bioinformatics will improve the longitudinal integration of transcriptomic, proteomic and lipidomic data with clinical parameters. These analyses will help to increase the understanding of the complex interaction of internal and external variables during development, resulting in a healthy individual or one with programmed disease. While the strength of animal models lies in the possibility to elucidate molecular mechanisms, it will be a challenge for clinicians to identify individuals at risk. Harmonization and standardization of cohorts like in the LifeCycle-Project (293) improves the epidemiological basis to translate hypotheses on the early origins of disease from animal models in to the human context and to confirm their clinical relevance. In this context, research on biomarkers is of high relevance to improve the diagnostic options in early detection of aberrant organ development. Candidates have been studied in clinical situations of known organ damage, e.g., BDP (294, 295) and neonatal kidney injury (296–298) and will have to be tested in the context of metabolic programming. Importantly, the goal is not to label an individual organ function as pathological beyond classical criteria but to identify individuals who are at risk to develop disease later in life in order to provide targeted prevention strategies.

Looking at molecular mechanisms, it is important to note that early metabolic origins of disease are based on a complex encounter of small-scale dysregulations rather than one single dysregulated pathway. Interestingly, apparently distinct causes of nutritional programming can cause similar molecular alterations. As discussed in detail for the lung, both IUGR (299) and maternal obesity-associated (300) models seem to induce inflammation in other organs as well. Briefly summarized, it is demonstrated that neuroinflammation is an important mechanism contributing to neurocognitive impairment after IUGR and maternal obesity (301, 302) and the window of vulnerability extends well-beyond birth (303, 304). Circulating inflammatory proteins were even tested as biomarkers for later cognitive impairment in preterm infants (305). Experimental studies have also linked perinatal inflammation to adverse kidney development (306, 307) and cardiac dysfunction (308). Taken together, these studies highlight the need to consider inter-organ communication as an important contributor to health and disease.

## Conclusion

**Figure 3:**
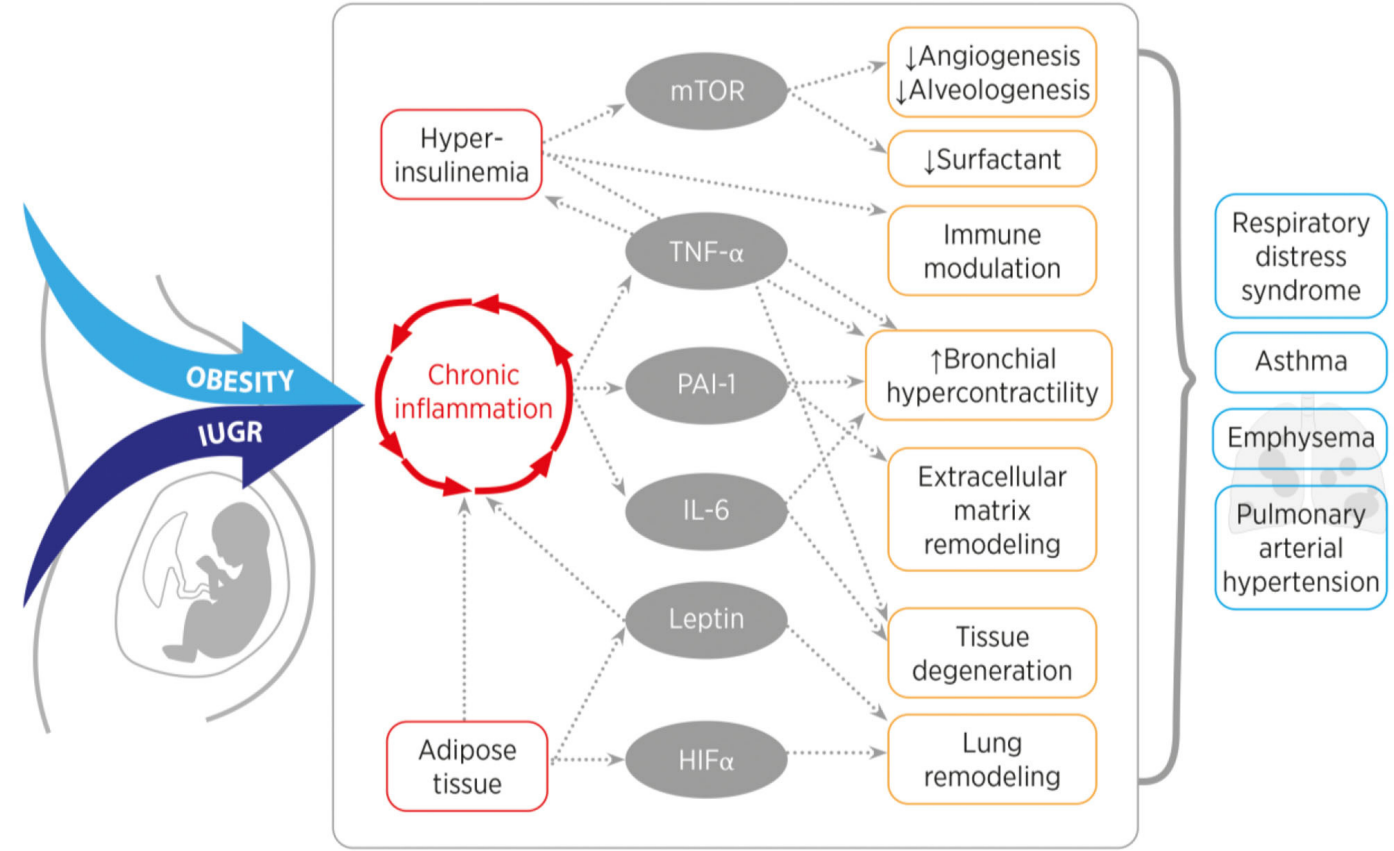
Overview of the converging inflammatory signaling and nutrient sensing pathways of obesity and intrauterine growth restriction (IUGR). Obesity or IUGR lead to chronic inflammation and hyperinsulinemia, which induces mTOR, TNF-α, PAI-1, IL-6, Leptin, and HIF-α signaling. In the lung, these signaling molecules cause for example tissue remodeling, reduce alveolarization and induce smooth muscle cell hyperreactivity. These features are characteristics for a higher susceptibility to develop a chronic lung disease in later life. [mTOR (mechanistic target of rapamycin), TNF-α (tumor necrosis factor alpha), PAI-1 (plasminogen activator inhibitor-1), IL-6 (interleukin-6), HIF-α (hypoxia inducible hypoxia-inducible factor alpha)].

The mission of this article was to provide a comprehensive review of the impact of perinatal nutrition and metabolism on lung development and early origins of CLD. The current literature provides compelling evidence that maternal obesity, early childhood obesity and IUGR are intimately linked to increased risk for lung disease. These perinatal nutritional alterations of the fetus and infant converge in similar metabolic, endocrine, nutrient sensing and inflammatory signaling pathways. Key regulators are insulin and leptin, and their respective downstream signaling cascades. Both hormones are essential for physiological growth and development during pregnancy; in contrast, interruption of the concerted interaction and balance of hormones, cytokines and growth factors during a critical window of development can disrupt developmental processes and adversely affect child (lung) health throughout life. For example, maternal obesity and early childhood obesity cause hyperinsulinemia and hyperleptinemia combined with insulin- and leptin-insensitivity. On the other hand, IUGR is characterized by a transient prenatal downregulation of insulin- and leptin signaling, followed by a postnatal upregulation during catch-up growth resulting in the same pathology as obesity. These two endocrine factors subsequently cause a cascade of pro-inflammatory programing with the release of (adipo-) cytokines and can contribute to metabolic and pulmonary disease (Figure 3, Table 1). These pulmonary sequelae span from aberrant alveolarization and angiogenesis to remodeling of the extracellular matrix and ultimately reducing lung function. While the present review primarily focused on the lung, other organs are affected as well, highlighting the importance of inter-organ communication. Beyond the metabolo-inflammatory stress response after perinatal nutritional alterations, smoking, air pollution as well as a consecutive dysbiosis of the intestinal and pulmonary microbiome contribute to the susceptibility and early origins of CLDs such as COPD, PAH and Asthma.

**Table 1:** Overview of the signaling molecules and pathways involved in the perinatal nutritional and metabolic origins of chronic lung diseases.

**Disease**	**Regulator**	**Obesity**	**IUGR**	**Effector**	**Outcome and reference**	
Pulmonary arterial hypertension (PAH)	HIF-α ↓	✓		Endothelin-1 ↑	Vascular remodeling (65, 66)	
	IL-6 ↑	✓	✓	Stat3 ↑ FoxO1 ↓	SMC proliferation (92, 93, 178)	
	PPARγ ↓	✓			Reduced SMC proliferation (130, 131)	Protective
	mTOR ↓		✓	VEGF ↓ BMP ↓	Reduced angiogenesis Altered ECM disposition (157, 218)	
COPD and emphysema	IL-6 ↑	✓	✓		ATII apoptosis (91, 232)	
	Leptin			Sftpa ↑	ATII maturation (104, 105)	Protective
	↑	✓	✓	Col1a1, Col3a1, Col6a3, Mmp2, Tieg1, Stat1 ↑	Enlarged alveoli (106, 157)	
Respiratory distress syndrome (RDS)	Insulin ↑	✓	✓	VEGF ↓ HIF-2 ↓ mTOR ↑	Reduced angiogenesis (141)	
		✓	✓	PI3K ↑ Sftpa ↓	Increased alveolar surface tension (139)	
			✓	GH/IGF-1 ↓ ↑	Reduced alveologenesis Lung- and bodygrowth (174, 204)	Protective
	PPARγ				Promotes Lung maturation (128, 129)	Protective
	Leptin ↓	✓	✓	Leptin resistance ↑ mTOR ↓	Reduced alveolar surface (173, 210, 213, 214)	
Asthma	TNF-α ↑	✓	✓	G-proteins ↑	Hyperreactivity in SMC (96, 232)	
	Adiponectin ↓	✓		NF-κB ↑	Enhanced TNF-α activity (97)	
	PAI-1 ↑	✓			Collagen, fibrin deposition (89)	
	Insulin ↑	✓	✓	Th2 shift	Enhanced immune response (145)	
		✓	✓	PI3K-signaling ↑	Contractile SMC phenotype (143)	
	Leptin ↑	✓	✓	mTOR ↑ MAPK ↑	Hyperreactivity (114)	

Both obesity-related comorbidities and CLDs are a relevant socioeconomic and individual burden. The alarmingly increasing rates of overweight and obese adults, pregnant women, and children emphasize the need to investigate and decipher the crosstalk between nutrition metabolism and the early origins of CLDs. Elucidation of the metabolo-pulmonary axis with subsequent identification of novel targets will provide new avenues to prevent metabolic programming and the early origins of CLDs.

## Author Contributions

CK-M, JS, EN, and MA conceived, designed, and drafted the manuscript. CK-M, JS, EN, JD, and MA edited and revised the manuscript and approved the final version of the manuscript. All authors contributed to the article and approved the submitted version.

## Conflict of Interest

The authors declare that the research was conducted in the absence of any commercial or financial relationships that could be construed as a potential conflict of interest.
